# Modifiable risk factors for overweight and obesity in children and adolescents from São Paulo, Brazil

**DOI:** 10.1186/1471-2458-11-585

**Published:** 2011-07-22

**Authors:** Scott Duncan, Elizabeth K Duncan, Romulo A Fernandes, Camila Buonani, Karolynne D-N Bastos, Aline FM Segatto, Jamile S Codogno, Igor C Gomes, Ismael F Freitas

**Affiliations:** 1 Centre for Physical Activity and Nutrition, Auckland University of Technology, Auckland, New Zealand; 2 Department of Physical Education, São Paulo State University, São Paulo, Brazil

## Abstract

**Background:**

Brazil is currently experiencing a nutrition transition: the displacement of traditional diets with foods high in saturated fat, sodium, and cholesterol and an increase in sedentary lifestyles. Despite these trends, our understanding of child obesity in Brazil is limited. Thus, the aims of this study were (1) to investigate the current prevalence of overweight and obesity in a large sample of children and adolescents living in São Paulo, Brazil, and (2) to identify the lifestyle behaviors associated with an increased risk of obesity in young Brazilians.

**Methods:**

A total of 3,397 children and adolescents (1,596 male) aged 7-18 years were randomly selected from 22 schools in São Paulo, Brazil. Participants were classified as normal weight, overweight, or obese based on international age- and sex-specific body mass index thresholds. Selected sociodemographic, physical activity, and nutrition behaviors were assessed via questionnaire.

**Results:**

Overall, 19.4% of boys and 16.1% of girls were overweight while 8.9% and 4.3% were obese. Two-way analysis of variance revealed that the prevalence of overweight and obesity was significantly higher in boys and in younger children when compared to girls and older children, respectively (P < 0.05 for both). Logistic regression analysis revealed that overweight was associated with more computer usage, parental encouragement to be active, and light soft drink consumption after controlling for differences in sex, age, and parental education (P < 0.05 for all). Conversely, overweight was associated with less active transport to school, eating before sleep, and consumption of breakfast, full-sugar soft drinks, fried food and confectionery (P < 0.05 for all).

**Conclusions:**

Our results show that obesity in São Paulo children and adolescents has reached a level equivalent to that seen in many developed countries. We have also identified three key modifiable factors related to obesity that may be appropriate targets for future intervention in Brazilian youth: transport mode to school, computer usage, and breakfast consumption.

## Background

Obesity in children and adolescents is widely considered to be a worldwide crisis, not only due to its harmful physiological effects, but also for its negative impact on quality of life [1]. The majority of research to date has focused on child obesity in the developed world. However, child obesity is also a serious concern in developing countries experiencing a nutrition transition: the displacement of traditional diets with foods high in saturated fat, sodium, and cholesterol and an increase in sedentary lifestyles. In Brazil, the prevalence of youth obesity rose from 4.1% in 1975 to 13.9% in 1997 [2]. Indeed, obesity has now surpassed malnutrition as the most prevalent nutritional disorder in Brazilian children. Non-representative studies indicate that the impact of child obesity in Brazil varies according to region: recent estimates range from 4.4% [3], 4.5% [4], and 8.5% [5] in the less developed Northeastern regions to 10.5% [6], 12.8% [7], and 18.0% [8] in the more developed Southeast (as defined by the Instituto Brasileiro de Geografia e Estatística [9]). Given this level of diversity, it is clear that the child obesity issue in Brazil must be addressed at the regional rather than the national level.

To our knowledge, only three representative studies have investigated obesity in school-aged children from São Paulo, Brazil's most populace state (over 40 million inhabitants). In 2000, da Costa Ribeiro et al [6] measured the height and weight of 2,519 children aged 7-10 years and found that 10.5% were classified as obese. In this instance, obesity was defined as a height-to-weight greater than or equal to two standard deviations above the median of a World Health Organization reference population. More recently, we applied the age- and sex-specific body mass index (BMI) cut-off points established by the International Obesity Taskforce (IOTF) [10] - the most widely used definition of youth obesity - to two independent samples of young people from the city of Presidente Prudente in São Paulo State. In the first study, we found that 28.6% of 1,215 youth aged 10-17 years were classified as overweight/obese [11]. In the second study, only 22.9% of adolescents aged 11-18 years were overweight/obese [12]. Given the present transition from under-nutrition to over-nutrition, it is vital that we continue to monitor trends in child obesity within Brazil's most populace regions using internationally applicable classification systems.

Nevertheless, identifying the scale of the problem is only the first step towards developing appropriate preventative strategies. In order to design effective interventions we need an understanding of the modifiable risk factors associated with fat accretion in children. Several studies have identified key sociodemographic correlates of obesity in Brazilian children, such as sex [5,8,11,12], age [2,5,13], socioeconomic status [4,14], ethnic group [3,14], familial characteristics [14], parental education [12], and school type [3,4,8,12]. While it is important to determine which groups to target, none of the identified factors are modifiable through lifestyle intervention. To our knowledge, only two studies have investigated modifiable risk factors related to child obesity in Brazil: the aforementioned study by da Costa Ribeiro et al [6] conducted in São Paulo children ten years ago and a smaller but more recent study of 511 Porto Alegre children and adolescents aged 10-18 years [13]. The former authors identified two behavioral characteristics that were positively associated with obesity in children: appetite at meals (odds ratio: 3.67) and excessive television watching (odds ratio: 1.89). The latter authors reported that obese children/adolescents ate relatively fewer meals each day and consumed more diet soft drinks (chi-squared tests, P < 0.05).

It is clear that our existing knowledge of obesity and its determinants in Brazilian youth is lacking. Thus, the aims of this study were (1) to investigate the current prevalence of overweight and obesity in a large sample of children and adolescents living in São Paulo, Brazil, and (2) to identify the lifestyle behaviors associated with an increased risk of obesity in young Brazilians.

## Methods

### Participants

A total of 3,397 children and adolescents (1,596 male, 1,801 female) aged 7-18 years were randomly selected from 11 public and 11 private schools in the state of São Paulo, Brazil. Participating schools were purposively sampled from 10 small (< 50,000 inhabitants) to medium sized (50,000-500,000 inhabitants) cities in the west region of São Paulo state to include both ends of the socioeconomic distribution. There are 55 schools in total within these cities. Children were given information about the study and invited to participate within class. If a child did not wish to participate, the next child of the same sex and age on a randomly generated list was invited. Each participant was required to obtain written, informed consent from his or her legal guardian before they could take part in the measurement procedures. The overall consent rate was 90%, suggesting that sampling bias is unlikely to be present. The ethnic composition of the sample was 74.1% white, 15.2% black, and 10.8% Asian. Ethical approval for this study was obtained from the Universidade Estadual Paulista ethics committee (087/2006).

### Procedures

Height was measured to the nearest 0.1 cm with a portable stadiometer, and weight was measured in light clothing to the nearest 0.1 kg on a digital scale (Filizola, São Paulo, Brazil). All anthropometric data were collected by trained staff. BMI was calculated as weight (kg) divided by squared height (m^2^). Participants were classified as either non-overweight or overweight according to international sex- and age-specific BMI cut-off points [10].

Data pertaining to lifestyle habits were collected via a self-report questionnaire administered immediately prior to anthropometric measurements. Questions were designed to assess behaviors related to physical activity (mode of transport to school, sports participation, parental sports participation, sibling activity, and parental encouragement to be active), sedentary habits (television watching, computer usage, and homework patterns), and dietary practices (breakfast consumption, food purchased at school, eating before sleep, and consumption of soft drinks [full sugar and light], fruit juice, fried food, confectionary, fruits and vegetables). Potential answers for the sports, television, computer, and homework questions were 'none', '<1 hr/day', '1-2 hr/day', and '>2 hr/day'. Answers for the breakfast, school lunch, eating before sleep, and soft drink, fruit juice, fried food, and confectionery questions were 'never', '1-2 days/week', '3-5 days/week', and '>5 days/week'. Answers for fruit and vegetable consumption were 'never', '<1 portion/day', '1-2 portions/day', and '>2 portions/day'. One portion of fruit was defined as 'one medium piece or two small pieces', and one portion of vegetables was defined as 'half a cup of cooked vegetables or one cup of salad'. The wording of the questionnaire was developed in consultation with a group of children and adolescents from the target population (7-18 years). In our pilot work, the questionnaire showed satisfactory levels of acceptability, ease of completion, reliability, and content validity. All data were collected during morning and afternoon class periods.

### Statistical analyses

Differences in participant characteristics (age, height, weight, BMI) between sexes and among age groups were assessed by two-way ANOVA, with significant associations examined by pairwise comparisons using t-tests. Differences in the prevalence of overweight and obesity were examined using chi-squared analysis. Logistic regression analysis was used to investigate associations between overweight/obesity and the selected sociodemographic, physical activity, and dietary variables. Odds ratios for each category were adjusted for sex, age, father education, mother education. All data were analyzed using SPSS 16 (SPSS Inc., Chicago, IL) with a P value less than 0.05 used to indicate statistical significance.

## Results

Table 1 shows the physical characteristics of the study sample grouped according to age and sex. Significant differences in height, weight and BMI were detected across age groups for both sexes. Although 15-18-year-old boys were heavier than girls from the same age group, there were no significant sex differences in BMI. Boys were taller, heavier, and had a greater BMI than girls when all age groups were combined.

**Table 1:** Participant characteristics; results are mean ± SD.

	**7-10 yr**				**11-14 yr**				**15-18 yr**				**All**			
	**Male (n = 317)**		**Female (n = 340)**		**Male (n = 796)**		**Female (n = 823)**		**Male (n = 483)**		**Female (n = 638)**		**Male (n = 1,596)**		**Female (n = 1,801)**	
	**Mean**	**SD**	**Mean**	**SD**	**Mean**	**SD**	**Mean**	**SD**	**Mean**	**SD**	**Mean**	**SD**	**Mean**	**SD**	**Mean**	**SD**
Age (yr)	9.4	1.2	9.4	1.2	13.0^†^	1.1	12.9^†^	1.1	16.4^‡^	0.8	16.4^‡^	0.7	13.3	2.6	13.5	2.7
Height (cm)	137.1	9.6	137.2	10.1	157.8^†^	11.3	156.3^†^	7.8	171.6^‡^	7.8	162.6^‡^	6.5	158.9	15.5	155.6^§^	18.5
Weight (kg)	35.5	10.2	33.8	99	51.5^†^	15.1	48.5^†^	11.1	63.2^‡^	11.9	55.8^‡§^	9.7	51.9	16.3	48.6^§^	12.9
BMI (kg.m^-2^)	18.6	3.8	17.6	3.4	20.3^†^	4.1	19.7^†^	3.6	21.4^‡^	3.3	21.0^‡^	3.4	20.3	4.0	19.9^§^	3.7

^†^Signficantly different from 7-10 yr of same sex (P < 0.001). ^‡^Signficantly different from 11-14 yr of same sex (P < 0.001). ^§^Significantly different from male of same age group (P < 0.001).

**Figure 1:**
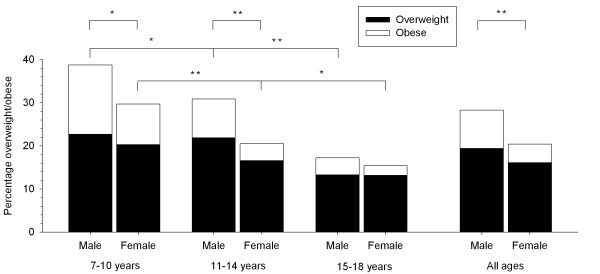
**Prevalence of overweight and obesity in São Paulo children and adolescents aged 7-18 years**.  *Significant difference (P < 0.05). **Significant difference (P < 0.01).

Figure 1 shows the prevalence of overweight and obesity in each age and sex group. Overall, 19.4% of boys and 16.1% of girls were overweight with a further 8.9% of boys and 4.3% of girls classified as obese. The prevalence of overweight and obesity decreased significantly as age group increased in both boys and girls. Significant sex differences were also observed, with boys showing a greater prevalence of overweight and obesity than girls in those aged 7-10 and 11-14 years and across the whole sample. In addition, the ratio of overweight to obesity in each sex and age group varied considerably, ranging from 1.4:1 in 7-10-year-old boys to 6:1 in 15-18-year-old girls. The prevalence of underweight - defined using the World Health Organization BMI-for-age curves - was 3.5% in boys and 3.9% in girls.

Table 2 shows the unadjusted and adjusted (sex, age, father education, mother education) odds ratios for overweight and obesity for each of the sociodemographic variables assessed. Both sex and age remained significant in the adjusted model. The odds of overweight/obesity were 0.65 times lower in girls than in boys, and 0.66 and 0.36 times lower in those aged 11-14 and 15-18 years (respectively) when compared with 7-10-year-olds.

**Table 2:** Sociodemographic correlates of overweight and obesity in São Paulo children and adolescents aged 7-18 years.

	**Number of participants (%)**		**Unadjusted Odds Ratio (95% CI)**	**Adjusted Odds Ratio (95% CI)^†^**
	**Normal weight**	**Overweight/obese**		
Sex				
Male	1,144 (44.4%)	452 (55.1%)	1.00	1.00
Female	1,433 (55.6%)	368 (44.9%)	0.65 (0.55-0.76)**	0.65 (0.55-0.77)**
Age				
7-10 yr	433 (16.8%)	224 (27.3%)	1.00	1.00
11-14 yr	1,204 (46.7%)	415 (50.6%)	0.67 (0.55-0.81)**	0.66 (0.54-0.81)**
15-18 yr	940 (36.5%)	181 (22.1%)	0.37(0.30-0.47)**	0.36 (0.29-0.46)**
Father education				
No education	34 (1.4%)	10 (1.3%)	1.00	1.00
Primary	647 (26.5%)	207 (27.0%)	1.09 (0.53-2.24)	0.82 (0.38-1.80)
Secondary	722 (29.6%)	237 (30.9%)	1.12 (0.54-2.29)	0.80 (0.36-1.75)
College	1,038 (42.5%)	312 (40.7%)	1.02 (0.50-2.09)	0.69 (0.31-1.52)
Mother education				
No education	42 (1.7%)	9 (1.2%)	1.00	1.00
Primary	686 (28.1%)	217 (28.3%)	1.48 (0.71-3.08)	1.52 (0.69-3.35)
Secondary	665 (27.2%)	219 (28.6%)	1.54 (0.74-3.21)	1.58 (0.71-3.50)
College	1,048 (42.9%)	321 (41.9%)	1.43 (0.69-2.97)	1.64 (0.73-3.69)

^†^Adjusted for sex, age, father education, and mother education. *Significantly different from reference group (P < 0.05). **Significantly different from reference group (P < 0.01).

Table 3 shows the unadjusted and adjusted odds ratios for overweight and obesity for each of the variables related to physical activity and sedentary behavior. Four variables showed significant associations with the prevalence of overweight/obesity (travel to school, computer usage, parental sport, parental encouragement); all remained significant after adjustment for sociodemographic variables. Compared with those who were driven to school by car, the odds of overweight/obesity were 0.72, 0.61, and 0.59 times lower in participants who bussed, cycled, and walked to school, respectively. The odds of overweight/obesity were 1.64 and 1.94 times higher in participants who spent 1-2 and > 2 hours/day on the computer when compared to those who did not use the computer at all. Additionally, those with one parent involved in sport showed 1.25 times greater odds of overweight/obesity than those with no sporting parents, while those who received encouragement to be active from one and both of their parents showed 1.67 and 1.63 greater odds of overweight/obesity (respectively) than those who received no encouragement from parents.

**Table 3:** Physical activity correlates of overweight and obesity in São Paulo children and adolescents aged 7-18 years.

	**Number of participants (%)**		**Unadjusted Odds Ratio (95% CI)**	**Adjusted Odds Ratio (95% CI)^†^**
	**Normal weight**	**Overweight/obese**		
Travel to school				
Car	846 (35.2%)	352 (46.3%)	1.00	1.00
Bus	357 (14.8%)	118 (15.5%)	0.79 (0.62-1.01)	0.72 (0.56-0.93)*
Bicycle	122 (5.1%)	37 (4.9%)	0.73 (0.49-1.08)	0.61 (0.40-0.94)*
Walk	1,044 (43.4%)	234 (30.8%)	0.54 (0.45-0.65)**	0.59 (0.48-0.72)**
Other	37 (1.5%)	19 (2.5%)	1.23 (0.70-2.18)	1.32 (0.68-2.57)
Sport				
None	730 (30.3%)	206 (27.1%)	1.00	1.00
1-2 days/week	796 (33.1%)	270 (35.5%)	1.20 (0.98-1.48)	1.22 (0.98-1.52)
3-4 days/week	541 (22.5%)	173 (22.8%)	1.13 (0.90-1.43)	1.07 (0.83-1.37)
> 4 days/week	339 (14.1%)	111 (14.6%)	1.16 (0.89-1.51)	1.01 (0.76-1.35)
Television				
None	58 (2.4%)	17 (2.2%)	1.00	1.00
< 1 hr/day	421 (17.5%)	131 (17.2%)	1.06 (0.60-1.89)	0.99 (0.55-1.81)
1-2 hr/day	989 (41.1%)	341 (44.9%)	1.18 (0.68-2.05)	1.19 (0.67-2.12)
> 2 hr/day	938 (39.0%)	271 (35.7%)	0.99 (0.57-1.72)	0.90 (0.50-1.60)
Homework				
None	162 (6.7%)	46 (6.1%)	1.00	1.00
< 1 hr/day	1,255 (52.2%)	394 (51.8%)	1.11 (0.78-1.56)	1.00 (0.70-1.45)
1-2 hr/day	774 (32.2%)	267 (35.1%)	1.22 (0.85-1.73)	1.33 (0.92-1.94)
> 2 hr/day	215 (8.9%)	53 (7.0%)	0.87 (0.56-1.35)	0.95 (0.59-1.51)
Computer				
None	746 (31.0%)	181 (23.8%)	1.00	1.00
< 1 hr/day	369 (15.3%)	112 (14.7%)	1.25 (0.96-1.63)	1.32 (0.99-1.75)
1-2 hr/day	624 (25.9%)	206 (27.1%)	1.36 (1.09-1.71)*	1.64 (1.28-2.09)**
> 2 hr/day	667 (27.7%)	261 (34.3%)	1.61 (1.30-2.00)**	1.94 (1.54-2.45)**
Parental sport				
None	1,388 (57.7%)	405 (53.3%)	1.00	1.00
One	564 (23.4%)	208 (27.4%)	1.26 (1.04-1.53)*	1.25 (1.02-1.53)*
Both	454 (18.9%)	147 (19.3%)	1.11 (0.89-1.38)	1.04 (0.83-1.31)
Parental encouragement				
None	493 (20.5%)	113 (14.9%)	1.00	1.00
One	487 (20.2%)	168 (22.1%)	1.51 (1.15-1.97)*	1.67 (1.26-2.32)**
Both	1,426 (59.3%)	479 (63.0%)	1.47 (1.17-1.84)*	1.63 (1.27-2.08)**
Active sibling(s)				
Yes	1,187 (49.3%)	356 (46.8%)	1.00	1.00
No	1,219 (50.7%)	404 (53.2%)	1.11 (0.94-1.30)	1.10 (0.92-1.30)

^†^Adjusted for sex, age, father education, and mother education. *Significantly different from reference group (P < 0.05). **Significantly different from reference group (P < 0.01).

Table 4 shows the unadjusted and adjusted odds ratios for overweight and obesity for each of the dietary variables. Five variables showed significant associations with overweight/obesity in both models (breakfast, eating before sleep, soft drinks [full], soft drinks [light], confectionery), while one variable was significant for the adjusted model only (fried food). The odds of overweight/obesity were 0.59 times lower in participants who ate breakfast on more than five days in the previous week than those who did not eat breakfast. Eating before sleep and soft drink (full), fried food, and confectionery consumption had similar effects on the odds of overweight/obesity, whereas the consumption of light soft drinks was associated with increased odds of overweight/obesity.

**Table 4:** Dietary correlates of overweight and obesity in São Paulo children and adolescents aged 7-18 years.

	**Number of participants (%)**		**Unadjusted Odds Ratio (95% CI)**	**Adjusted Odds Ratio (95% CI)^†^**
	**Normal weight**	**Overweight/obese**		
Breakfast				
Never	264 (10.8%)	127 (16.6%)	1.00	1.00
1-2 days/week	293 (12.0%)	116 (15.1%)	0.82 (0.61-1.11)	0.93 (0.68-1.27)
3-5 days/week	234 (9.6%)	73 (9.5%)	0.65 (0.46-0.91)*	0.73 (0.52-1.03)
> 5 days/week	1,650 (67.6%)	450 (58.7%)	0.57 (0.45-0.72)**	0.59 (0.46-0.75)**
Buy school food				
Never	946 (38.8%)	287 (37.5%)	1.00	1.00
1-2 days/week	773 (31.7%)	268 (35.0%)	1.14 (0.94-1.38)	1.21 (0.99-1.47)
3-5 days/week	337 (13.8%)	99 (12.9%)	0.97 (0.75-1.26)	1.06 (0.81-1.39)
> 5 days/week	385 (15.8%)	112 (14.6%)	0.96 (0.75-1.23)	0.99 (0.77-1.28)
Eat before sleep				
Never	887 (36.3%)	382 (49.9%)	1.00	1.00
1-2 days/week	489 (20.0%)	135 (17.6%)	0.64 (0.51-0.80)**	0.71 (0.57-0.90)*
3-5 days/week	283 (11.6%)	73 (9.5%)	0.60 (0.45-0.80)**	0.59 (0.44-0.78)**
> 5 days/week	782 (32.0%)	176 (23.0%)	0.52 (0.43-0.64)**	0.46 (0.38-0.57)**
Soft drinks (full)				
Never	301 (12.3%)	125 (16.3%)	1.00	1.00
1-2 days/week	840 (34.4%)	290 (37.9%)	0.83 (0.65-1.07)	0.82 (0.64-1.06)
3-5 days/week	647 (26.5%)	192 (25.1%)	0.72 (0.55-0.93)*	0.71 (0.54-0.92)*
> 5 days/week	653 (26.8%)	159 (20.8%)	0.59 (0.45-0.77)**	0.53 (0.40-0.70)**
Soft drinks (light)				
Never	1,668 (68.3%)	419 (54.7%)	1.00	1.00
1-2 days/week	495 (20.3%)	200 (26.1%)	1.61 (1.32-1.96)**	1.59 (1.31-1.95)**
3-5 days/week	151 (6.2%)	80 (9.8%)	2.11 (1.58-2.82)**	1.99 (1.48-2.68)**
> 5 days/week	127 (5.2%)	67 (8.7%)	2.10 (1.53-2.88)**	1.65 (1.19-2.29)*
Fruit juice				
Never	248 (10.2%)	73 (9.5%)	1.00	1.00
1-2 days/week	975 (39.9%)	269 (35.1%)	0.94 (0.70-1.26)	0.96 (0.71-1.30)
3-5 days/week	742 (30.4%)	237 (30.9%)	1.09 (0.81-1.46)	1.11 (0.82-1.51)
> 5 days/week	476 (19.5%)	187 (24.4%)	1.34 (0.98-1.82)	1.19 (0.86-1.63)
Fried food				
Never	249 (10.2%)	79 (10.3%)	1.00	1.00
1-2 days/week	946 (38.8%)	340 (44.4%)	1.13 (0.86-1.50)	0.98 (0.73-1.31)
3-5 days/week	789 (32.3%)	224 (29.2%)	0.90 (0.67-1.20)	0.76 (0.56-1.03)
> 5 days/week	457 (18.7%)	123 (16.1%)	0.85 (0.62-1.17)	0.64 (0.46-0.90)*
Confectionery				
Never	107 (4.4%)	52 (6.8%)	1.00	1.00
1-2 days/week	814 (33.3%)	323 (42.2%)	0.82 (0.57-1.17)	0.84 (0.58-1.21)
3-5 days/week	797 (32.7%)	222 (29.0%)	0.57 (0.40-0.82)*	0.56 (0.38-0.81)*
> 5 days/week	723 (29.6%)	169 (22.1%)	0.48 (0.33-0.70)**	0.43 (0.29-0.63)**
Fruit				
Never	196 (8.0%)	59 (7.7%)	1.00	1.00
< 1 portion/day	1,043 (42.7%)	327 (42.7%)	1.04 (0.76-1.43)	1.20 (0.86-1.65)
≥ 1 portion/day	1,202 (49.2%)	380 (49.6%)	1.05 (0.77-1.44)	1.12 (0.81-1.54)
Vegetables				
Never	316 (12.9%)	106 (13.8%)	1.00	1.00
< 1 portion/day	881 (36.1%)	263 (34.3%)	0.89 (0.69-1.15)	1.01 (0.77-1.32)
≥ 1 portion/day	1,244 (51.0%)	397 (51.8%)	0.95 (0.74-1.22)	1.01 (0.79-1.30)

^†^Adjusted for sex, age, father education, and mother education. *Significantly different from reference group (P < 0.05). **Significantly different from reference group (P < 0.01).

## Discussion

The rising prevalence of childhood obesity in Brazil is a serious public health dilemma given its established link to chronic health disorders such as dyslipidemia and hypertension [7,15]. Given the regional variance in previous estimates of obesity in Brazilian youth [3-8], it is essential that we monitor the obesity trends in key populations. The present study represents the largest investigation of overweight and obesity in São Paulo children and adolescents. Our findings showed that obesity in São Paulo youth has reached a critical stage, with 28.3% of boys and 20.4% of girls either overweight or obese. These totals are comparable with youth overweight data from developed countries such as the UK (23.6% in boys, 27.9% in girls) [16], Australia (25.8% in boys, 24.0% in girls) [17], and New Zealand (29.2% in both boys and girls) [18], but are still several percentage points lower than American estimates (35.3% in boys, 34.1% in girls) [19]. Data from the late 1990s showed that Brazilian youth, as in many developing countries, had begun to experience a nutritional transition from traditional diets and high levels of activity to high-fat diets and sedentary lifestyles, particularly in urban centers [2]. These trends have accompanied a rapid emergence from poverty: Brazil's per capita gross domestic product increased from less than $1,000 in the mid 1970s to more than $8,200 in 2008 [20]. It is important to note, however, that the prevalence of childhood overweight and obesity in many developed countries appears to be stabilizing. In contrast, the relatively high obesity estimates presented here (compared with previous national and regional data) suggest that the nutritional transition may not have reached a conclusion in Brazil. It is therefore imperative that public and community health programs for preventing obesity in Brazilian youth are given at least as much weight as those aiming to avert undernutrition.

Our data also revealed significant associations between obesity and both age and sex. The substantial decrease in overweight and obesity with age was unexpected given that surveys in developed countries have reported either stable [18,19] or positive [16,17] trends. The prevailing perception is that older children have had more time exposed to an obesogenic environment and more years with which to accumulate fat. Our results contradict this theory by suggesting that older children are at considerably less risk of overweight and obesity than younger children. It is possible that Brazilian youth, unlike those from developed countries, are subjected to unhealthier environments during early childhood when compared with late childhood and adolescence. Indeed, two other studies have reported the same age-related obesity trends in their respective samples of Brazilian children and adolescents [2,13]. The greater prevalence of overweight and obesity in boys compared with girls was less surprising given our previous work in this population [11,12]. Nonetheless, other research in Brazilian youth has been equivocal: some studies concurred that boys are more likely to be overweight than girls [5,8] while others were unable to find significant differences by sex [3,6,14]. The lack of association between child obesity and parental education was similar to our previous findings in São Paulo youth [12], indicating that this is not an important predictor of weight status in children. Nonetheless, it is clear that there are regional differences that influence the role of several sociodemographic factors on the risk of childhood obesity.

While it is clear that Brazilian youth are suffering from high levels of overweight and obesity, our understanding of the key modifiable risk factors responsible for current trends is lacking. Our findings identified several factors related to physical activity and dietary behavior. Participants who walked, cycled, or bussed to school were at a considerably lower risk of overweight and obesity than those who were driven to school by car. Walking, in particular, showed the strongest differences in the odds of overweight/obesity. A recent review on the topic concluded that while the link between active transport to/from school and overall physical activity levels in children from developed countries is relatively well established, the association between active transport and body composition in children is less compelling [21]. However, two more recent studies have posited an alternative viewpoint. Perhaps the most convincing evidence was provided by Pabayo et al [22], whose three-year longitudinal study showed that sustained active transport was associated with a favorable BMI trajectory over the early school years. In addition, Singh et al [23] concluded that a greater frequency of active transport to and from school partially explained the relatively low mean BMI observed in Dutch adolescents when compared to their non-Western counterparts. To our knowledge, the association between active transport and body composition has not been explored previously in children and adolescents from Brazil or any other developing country. Our data indicate that the promotion of active transport may be a key strategy in reducing the risk of youth obesity in Brazil. Further research is required to determine if this link exists in other developing countries.

The detection of a positive association between computer usage and the odds of overweight/obesity was notable, particularly given there were no associations between television usage and overweight/obesity. Previous research on sedentary habits in youth has focused primarily the television watching, with several studies showing positive effects of television on adiposity and obesity development [6,24-30]. A number of these studies also found no evidence of a relationship between computer usage and youth obesity [25,27,29,30], although one other study found that obesity was related to computer time and not to television time in Portuguese adolescents [31]. In recent years, the term 'screen time' has been introduced as an amalgamation of television, computer and any other time spent in front of an electronic screen (including mobile phones and portable gaming devices). Our data suggest that, at least in Brazil, not all sedentary screen-based activities have equivalent associations with child obesity. It is also of interest to note that computer usage was more frequent in adolescents than in younger children (even after adjustment for sociodemographic variables); this trend may help to explain the greater prevalence of overweight and obesity in older age groups. Regardless, we suggest that controlling the amount of time spend on a computer may be a more efficacious strategy than reducing television time, at least with regard to reducing youth obesity.

We also found that parental encouragement to be active and parental participation in sport were positively associated with overweight and obesity. The former trend can be explained by parents of overweight children attempting to prompt weight loss, a phenomenon observed in other populations [32,33]. The latter trend, however, is more difficult to explain. There is reasonable evidence that children of sporting or active parents are more likely to participate in active pursuits than those with non-sporting parents [34,35]. It is therefore logical to assume that parental activity would reduce the risk of obesity in their children, a theory supported by at least one study in American children [36]. Our observation that parental sport was associated with an increased risk of child obesity only when one parent (and not both) regularly took part in sport adds another layer of uncertainty. Clearly, more detailed investigation is required to elucidate the effects of parental participation in sport on the health status of their children.

Of the 10 dietary variables assessed, only breakfast frequency and consumption of light soft drinks followed expected trends. Skipping breakfast, in particular, appears to be a strong predictor of overweight in children and adolescents living in both developed [37,38] and developing [39,40] regions. While the mechanisms underlying this association are unclear, it is possible that skipping breakfast may lead to an up-regulation of appetite and poorer diet quality, ultimately resulting in weight gain over time. The association between light soft drink consumption and obesity may be explained by augmented weight loss practices in overweight children, including a shift from full sugar to 'diet' soft drinks. This trend has been observed previously in children and adolescents living in Porto Alegre, Brazil [13]; however, the practical implications for Brazilian public health are clearly limited.

Surprisingly, eating before sleep and the consumption of full sugar soft drinks, fried food, and confectionery showed negative associations with the odds of overweight/obesity in our sample. These findings were unanticipated given that such dietary practices are thought to increase the risk of childhood obesity in developed countries [37,41]. On the other hand, the only other study to compare the consumption of specific food groups with weight category in Brazilian youth reported that the habitual consumption of confectionary and fried foods were lower in obese participants when compared to those with a normal weight [13]. It is possible that, in Brazil, overweight children drastically reduce their intake of unhealthy foods and refrain from eating before sleep as a method of weight loss, or that these behaviors are imposed on them by their parents to a greater extent that in other countries. This would help to explain why older Brazilian children and adolescents are less likely to be obese than younger children when the opposite is generally observed in developed countries. It is also possible that obese Brazilian children are affected by response bias, providing answers that they perceive to be representative of a healthy diet. In any case, it appears that there are significant interactions between self-reported diet and obesity in Brazilian children and adolescents that do not necessarily present in other populations. These interactions should be taken into account when designing appropriate lifestyle interventions in this age group.

An obvious limitation of the present study is its cross-sectional design, which precludes statements of cause-and-effect. This becomes especially limiting when interpreting associations that could have two legitimate pathways, such as the negative association between obesity and walking to school. The extra activity accumulated via active transport may have a protective effect on the development of obesity, or it may simply be that obese children prefer to be driven to school. Until a longitudinal assessment of modifiable risk factors for obesity is conducted in Brazilian youth we must treat the present findings with caution. It is also possible that, given the relatively high number of comparisons, some of the significant associations reported in the present study may be chance findings rather than real relationships. Another limitation is that pubertal stage was not assessed. Given the wide age range of our sample, it is likely that puberty had a role to play in the onset of obesity and even in the selection of health behaviors. This is an area that could be explored in future research. Another potential limitation is that the questionnaire was only cognitive adaptive and the self-report questions pertaining to dietary and sedentary behaviors were not validated. While the questions were designed in conjunction with children to be age-appropriate in both language and content, we cannot rule out the potential presence of recall bias in younger children.

## Conclusions

In summary, we have demonstrated that obesity in São Paulo children and adolescents has reached a level equivalent to that seen in many developed countries. Furthermore, our results indicate that boys and younger children are more likely to be obese than girls and older children, respectively. We have also identified three key modifiable factors related to overweight that may be appropriate targets for future intervention in Brazilian youth: transport mode to school, computer usage, and breakfast consumption. Other factors that were associated with lower odds of overweight (eating before sleep, fried food and confectionery consumption) are likely to reflect increased weight loss efforts in obese children. Longitudinal evidence would provide further clarity regarding the effects of the selected risk factors during development.

## Competing interests

The authors declare that they have no competing interests.

## Authors' contributions

IFF conceived and designed the study. RAF, CB, KD-NB, AS, JC, and IG were responsible for recruitment, data collection, and data entry. SD and EKD analysed the data and drafted the manuscript. All authors contributed to writing and approved the final manuscript. The authors have no conflicts of interest to declare.

## Pre-publication history

The pre-publication history for this paper can be accessed here:

http://www.biomedcentral.com/1471-2458/11/585/prepub
